# Infections and antibiotics during fetal life and childhood and their relationship to juvenile idiopathic arthritis: a prospective cohort study

**DOI:** 10.1186/s12969-021-00611-4

**Published:** 2021-09-16

**Authors:** Erik Kindgren, Johnny Ludvigsson

**Affiliations:** 1 Department of Pediatrics, Skaraborg Hospital Skövde, SE-541 85 Skövde, Sweden; 2 Division of Pediatrics, Department of Biomedical and Clinical Sciences, Linköping University, Linköping, Sweden; 3 Division of Pediatrics, Department of Biomedical and Clinical Sciences, Linköping University, Crown Princess Victoria Children’s Hospital, Linköping, Sweden

**Keywords:** Juvenile idiopathic arthritis (JIA), Antibiotics, Infections, Arthritis, Epidemiology, Autoimmunity, Rheumatic disease

## Abstract

**Background:**

The aetiology of juvenile idiopathic arthritis (JIA) is poorly understood. It has been shown that use of antibiotics is associated with JIA. However, whether the association is due to increased occurrence of infection in these individuals is unknown. The purpose of this investigation was to measure the association between number of infections and use of antibiotics during childhood with development of JIA.

**Methods:**

In ABIS (All Babies in Southeast Sweden) a population-based prospective birth cohort of 17,055 children, data were collected on infections and antibiotic exposure during pregnancy and childhood. 102 individuals with JIA were identified. Multivariable logistic regression analyses were performed, adjusting for confounding factors.

**Results:**

Exposure to antibiotics during the periods 1–12 months, 1–3 years and 5–8 years was significantly associated with increased risk for JIA. The odds of developing JIA were three times higher in those exposed to antibiotics during the first 3 years of life compared with those not exposed (aOR 3.17; 95% CI 1.11–9.03, *p* = 0.031), and more than twice as high in those exposed to antibiotics during the first 5 years of life compared with those not exposed (aOR 2.18; 95% CI 1.36–3.50, *p* = 0.001). The odds of developing JIA were 78% higher in those exposed to antibiotics during the first 8 years of life compared with those not exposed (aOR 1.78; 95% CI 1.15–2.73, *p* = 0.009). Occurrence of infection during fetal life or childhood showed no significant association with the risk of developing JIA, after confounder adjustment.

The cumulative number of courses of antibiotics was significantly higher during childhood for the individuals who developed JIA (*p* < 0.001). Penicillins were more frequently used than non-penicillins, but both had an equal effect on the risk of developing JIA.

**Conclusions:**

Exposure to antibiotics early in life is associated with later onset of JIA in a large birth cohort from the general population. The relationship was dose dependent. These results suggest that further, more restrictive, antibiotic policies during the first years of life would be advisable.

## Background

The incidence of autoimmune diseases has increased over the last 50 years in parallel with an increased standard of living [1]. The underlying causes of JIA are mostly unknown [2]. Most rheumatic diseases are characterized by joint inflammation; however, inflammation can develop elsewhere in the body several years before the onset of joint inflammation [3–5]. Both juvenile idiopathic arthritis (JIA) and rheumatoid arthritis (RA) are commonly referred to as autoimmune diseases. Genetic components make up only a small part (10–25%) of the cause, and unknown environmental factors are believed to be the main cause of the disease [6, 7]. Environmental factors such as viral infections have been suggested, but other studies have not been able to confirm these results [8–13].

The intestinal microbiota is an environmental factor that affects metabolic and immunological homeostasis, maintains the integrity of the intestinal mucosa, and trains and contributes to the maturation of the immune system [14–17]. In a paediatric population whose immune system has not fully matured, changes in the intestinal microbiota may be more decisive for development of immune-mediated diseases than in adults. Thus, events in early life such as delivery method, diet, infections, and exposure to antibiotics may have a pronounced impact on the risk of developing autoimmune diseases in children. There is a great body of data indicating that antibiotic use, especially during childhood, is an important risk factor for development of atopy and inflammatory bowel disease [18]. Antibiotics are one of the most common prescription drugs in children and it seems to disrupt with the normal maturation of the microbiome [19–21]. A recent systematic review has compiled evidence that antibiotic exposure in children is associated with a reduction in wealth and / or diversity and a change in the balance between species in the microbiome with reductions in the number of commensal bacteria that are considered beneficial [22],

It has been shown that use of antibiotics is associated with both JIA and RA [23–25]. However, the association of is due to an increased number of infections, rather than use of the antibiotic, is unknown. In a large national register study with a case-control approach, it was noted that hospital care for infection in the first year of life was almost twice as common among those who later developed JIA [26]. A Finnish population study suggested that repeated childhood antibiotic exposure was associated with a subsequent diagnosis of JIA, but the study could not differentiate the effects of antibiotic use from the effects of infection [23]. Another study showed that antibiotics were associated with newly diagnosed JIA in a dose- and time-dependent manner in a paediatric population [24]. None of these studies had the opportunity to perform subgroup analyses for each JIA category to determine whether antibiotics were only associated with specific JIA manifestations.

To further elucidate the role of infections and/or use of antibiotics, the purpose of this study was to investigate the association between occurrence of infections and use of antibiotics during fetal life and childhood with later development of JIA.

## Methods

All parents of children born from October 1, 1997 to October 1, 1999 in south-eastern Sweden were asked to participate in ABIS (All Babies in South-east Sweden) [27, 28]. Of the 21,700 families surveyed, 17,055 (78.6%) participated. Blood samples have been taken from the children at birth (umbilical cord blood) and then at 1, 3, 5, and 8 years. Parental questionnaires containing information on nutrition, infections, drug use, vaccinations, and other factors were answered at birth, then at recurring intervals during childhood (including at 1, 3, 5 and 8 years of age). A primary purpose of the ABIS cohort is to identify the importance of environmental factors for development of immune-mediated diseases and elucidate how these genetic and environmental factors interact [27].

**Fig. 1:**
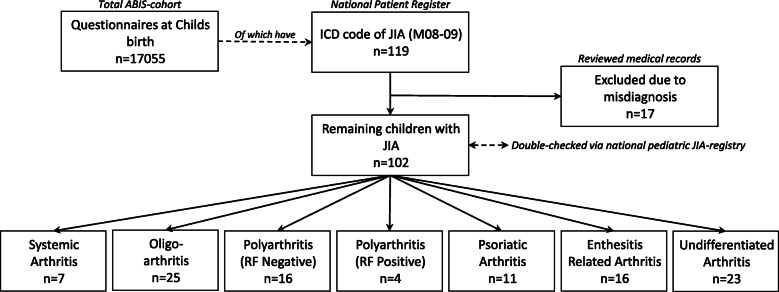
Flow-chart of the study sample, showing the number of total participants, patients with Juvenile Idiopathic Arthritis (JIA). ICD, International Classification of Diseases

To increase likelihood of detection for all patients with JIA, three methods were used to identify the patients. First, the parents indicated on a questionnaire at each follow-up whether the child was diagnosed with JIA. Second, data from the ABIS cohort was linked to the Swedish national patient register (https://www.socialstyrelsen.se/en/statistics-and-data/registers/register-information/the-national-patient-register/) with the help of the Swedish social security number, a unique 10-digit code [29]. Third, the diagnosis was validated upon review of the medical records for the patients, which resulted in some exclusions due to misdiagnosis (mostly monoarthritis, which later manifested as reactive arthritis). We identified 102 children with an International Classification of Diseases (ICD) code for JIA (ICD 9–10 code M08–M09) and who had agreed to participate in ABIS (see Fig. 1). Finally, all cases of JIA and their categories were cross-referenced via the Swedish JIA register (http://www.barnreumaregistret.se/), in which all pediatric rheumatologists in Sweden register and follow up their patients.

Exposure to infections and antibiotics was analysed from the questionnaires at birth, 1, 3, 5, and 8 years of age. At all age ranges, the questionnaire elicited the frequency of infections (otitis, tonsillitis, upper respiratory tract infection, pneumonia, meningitis, gastroenteritis, three-day fever, and influenza), as well as whether the child had experienced any infection that required antibiotic treatment. From the age of 3 years, the questionnaire also asked whether the child had been exposed to penicillin or other antibiotics (in this article referred to as non-penicillins).

Penicillin was explained in the questionnaires as a trademark for amoxicillin, phenoxymethylpenicillin, amoxicillin/clavulanic acid, while other antibiotics were explained as trademarks for trimethoprim, sulfamethoxazole, erythromycin, loracarbef, clarithromycin, cefadroxil, cefuroxime, clindamycin, among others.

### Statistical analysis

Multiple logistic regression was used to estimate the odds ratio (OR), with a 95% CI, for the significance of the explanatory variable. A *p*-value below 0.05 and a 95% CI not overlapping the null value 1.00 for the OR was regarded as statistically significant.

We adjusted our estimates for a wide range of confounding factors in a multiple regression model, generating an adjusted odds ratio (aOR). To investigate whether it is the underlying infection or course of antibiotics that is associated with JIA, these variables were adjusted against each other. Similarly, both infections and exposure to antibiotics were adjusted against breastfeeding, gestational age, and parental level of education in the final multiple regression model. Chi-square tests were used to assess the relationship between variables in basic characteristics. Statistical testing was corrected for false discovery rate (FDR) using the Benjamini & Hochberg method.

Statistics were calculated using IBM SPSS Statistics for Windows, version 25 (IBM Corp., Armonk, NY, USA).

## Results

A total of 17,055 children were included in the study, 102 of whom later received a JIA diagnosis during the follow-up period, which was 19 years. The median age at diagnosis was 12.0 years (1.9–16.0). Most cases were female (68%). The most common JIA category was oligoarthritis, followed by polyarthritis. The baseline characteristics of the study population are summarized in Table 1.

**Table 1:** Basic characteristics of cases and controls

	Controls	JIA	
	n (%)	n (%)	*p*
No. of subjects	16,369	102	
**Sex**			
Boys	8453 (52%)	33 (32%)	**< 0.001**
Girls	7814 (48%)	69 (68%)	
**Gestational age,** weeks (SD)	39.7 (2.4)	39.6 (2.0)	0.511
**Season of birth**			
Winter (December–February)	3840 (24%)	18 (18%)	0.155
Spring (March–May)	4607 (28%)	34 (33%)	0.268
Summer (June–August)	4270 (26%)	26 (25%)	0.854
Autumn (September–November)	3523 (22%)	24 (24%)	0.654
**Category at Onset (ILAR)** (n)			
**Systemic Arthritis**		7 (7%)	
**Oligoarthritis**		25 (25%)	
Persistent oligoarthritis		19 (19%)	
Extended oligoarthritis		6 (6%)	
**Polyarthritis (Rheumatoid Factor Negative)**		16 (16%)	
**Polyarthritis (Rheumatoid Factor Positive)**		4 (4%)	
**Psoriatic Arthritis**		11 (11%)	
**Enthesitis Related Arthritis**		16 (16%)	
**Undifferentiated Arthritis**		23 (23%)	

JIA = juvenile idiopathic arthritis; ILAR = International League of Associations for Rheumatology

*P*-value from chi-square test. Bold shows results reaching statistical significance (*p*-value below 0.05)

Infections, mainly reflecting bacterial infections, such as otitis during the first year of life was more frequent among cases than controls (39% vs 26%), as was tonsillitis in children aged 5–8 years (28% vs 15%), but after adjustment for potential confounders, including antibiotic treatment, the association no longer held significance. Other infections, mainly reflecting probable virus infections (upper respiratory tract infection, pneumonia, meningitis, gastroenteritis, three-day fever, and influenza) showed no statistical differences between cases and controls (Table 2).

**Table 2:** Number of infections during childhood in those who later developed JIA and in controls from the general population

	Control	JIA	aOR (95% CI)	p
**From 1 to 12 month**				
Upper respiratory tract infection (mean; SD)	3.7 (2.2)	3.9 (2.2)	OR 0.9 (0.6–1.2)	.382
Gastroenteritis	0.5 (0.8)	0.6 (0.8)	OR 1.5 (0.6–3.6)	.345
Influenza	0.2 (0.6)	0.3 (0.7)	OR 1.0 (1.0–1.0)	.995
**From 1 to 3 years**				
Common cold	5.3 (2.3)	5.5 (2.3)	OR 1.0 (0.8–1.4)	.810
Tonsillitis	0.3 (0.8)	0.3 (0.6)	OR 1.0 (1.0–1.0)	.994
Otitis	1.3 (1.8)	1.6 (1.9)	OR 1.3 (0.8–1.9)	.261
Pneumonia	0.1 (0.4)	0.2 (0.5)	OR 1.3 (0.3–6.0)	.764
Meningitis	0.0 (0.1)	0.0 (0.0)	OR 1.0 (1.0–1.0)	.999
Gastroenteritis	1.4 (1.3)	1.2 (1.0)	OR 0.5 (0.2–1.1)	.074
Three-day fever	0.6 (1.0)	0.6 (0.7)	OR 0.9 (0.4–1.9)	.845
Influenza	0.8 (1.2)	0.4 (0.8)	OR 0.6 (0.2–1.6)	.297
**From 3 to 5 years**				
Common cold	5.7 (2.3)	5.8 (2.1)	OR 1.0 (0.7–1.3)	.894
Tonsillitis	0.5 (1.1)	0.8 (1.5)	OR 0.9 (0.4–1.9)	.837
Otitis	1.2 (1.7)	1.3 (2.0)	OR 0.7 (0.4–1.4)	.331
Pneumonia	0.1 (0.3)	0.1 (0.4)	OR 2.2 (0.5–10.4)	.323
Meningitis	0.0 (0.2)	0.0 (0.0)	OR 1.0 (1.0–1.0)	.999
Gastroenteritis (viral)	2.1 (1.5)	2.4 (1.8)	OR 1.1 (0.7–1.7)	.556
Gastroenteritis (bacterial)	0.1 (0.4)	0.1 (0.3)	OR 1.0 (1.0–1.0)	.997
Pneumonia	0.1 (0.4)	0.1 (0.5)	OR 1.3 (0.3–6.5)	.748
Three-day fever	0.5 (1.0)	0.6 (1.4)	OR 1.1 (0.5–2.9)	.774
Influenza	0.9 (1.3)	1.3 (1.5)	OR 1.1 (0.6–2.2)	.693
**From 5 to 8 years**				
Common cold	4.0 (2.1)	4.6 (2.3)	OR 1.2 (0.9–1.7)	.252
Tonsillitis	0.3 (0.8)	0.5 (0.9)	OR 1.1 (0.4–2.6)	.899
Otitis	0.5 (1.1)	0.7 (1.4)	OR 1.0 (1.0–1.0)	.992
Meningitis	0.0 (0.2)	0.0 (0.0)	OR 1.0 (1.0–1.0)	.999
Gastroenteritis	1.4 (1.3)	1.7 (1.2)	OR 1.5 (0.8–2.5)	.181
Influenza	0.7 (1.0)	0.9 (1.1)	OR 1.0 (0.5–2.0)	.937

JIA = juvenile idiopathic arthritis; aOR = adjusted odds ratio (from logistic regression)

**Fig. 2:**
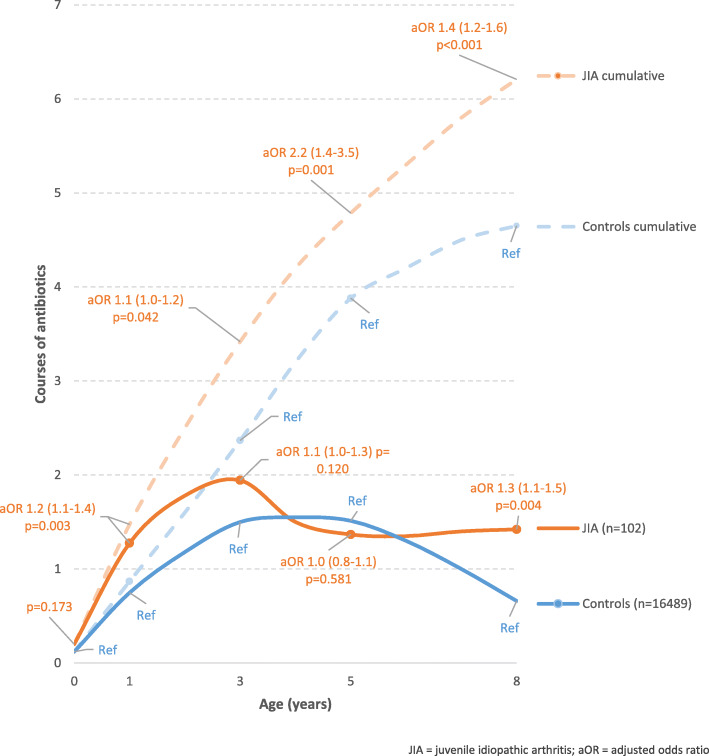
Number of doses of antibiotics (per time period) during childhood and cumulative number of antibiotic doses in children who later developed JIA, compared to controls from the general population

Exposure to antibiotics during the periods 1–12 months, 1–3 years and 5–8 years was significantly associated with a risk of JIA, when all JIA categories were analysed together (Table 3). The cumulative number of courses of antibiotics was also significantly higher during the first 8 years of life for the individuals who developed JIA (Fig. 2). Penicillins were used more frequently than non-penicillins, but both types of antibiotics had an equal effect on the risk of developing JIA (Table 3).

**Table 3:**
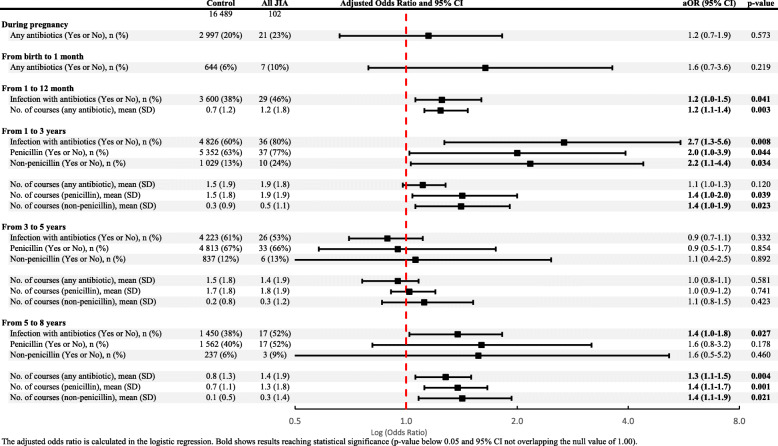
Exposure to antibiotics in the uterus and during the early childhood of those children who later developed JIA, compared to controls from the general population

The adjusted odds ratio is calculated in the logistic regression. Bold shows results reaching statistical significance (p-value below 0.05 and 95% CI not overlapping the null value of 1.00).

The odds of developing JIA were three times higher in those who had been exposed to antibiotics during the first 3 years of life compared with those who had not (aOR 3.17; 95% CI 1.11–9.03, *p* = 0.031). The corresponding odds of developing JIA were more than twice as high in those who had been exposed to antibiotics during the first 5 years of life compared with those who had not been exposed (aOR 2.18; 95% CI 1.36–3.50, *p* = 0.001). In addition, the odds of developing JIA were 78% higher in those who had been exposed to antibiotics during the first 8 years of life compared with those who had not been exposed (aOR 1.78; 95% CI 1.15–2.73, *p* = 0.009).

Since systemic-onset JIA (sJIA) is considered an autoinflammatory disease, it can be argued that this disease should not be assessed together with autoimmune diseases. All analyses in Table 3 were performed with sJIA (*n* = 7) excluded from the whole JIA group, and this did not change the result.

### Categories of JIA and antibiotic consumption

All categories of JIA and their relationship to antibiotic consumption during childhood are presented in Table 4. sJIA was the only category of JIA that had significantly higher antibiotic use during pregnancy (aOR 5.26; 95% CI 1.18–23.51, *p* = 0.030). Oligoarticular JIA showed significantly higher use of number of doses of penicillins at 5–8 years of age (aOR 1.44; 95% CI 1.03–2.02, *p* = 0.034).

**Table 4:** Exposure to antibiotics in the uterus and during the growth of those children who later developed JIA, categories of JIA compared to controls from the general population

	JIA																					
	**Control**	**sJIA**	**aOR (95% CI)**	**p**	**Oligo**	**aOR (95% CI)**	**p**	**Poly RF-**	**aOR (95% CI)**	**p**	**Poly RF+**	**aOR (95% CI)**	**p**	**PsA**	**aOR (95% CI)**	**p**	**ERA**	**aOR (95% CI)**	**p**	**Undiff.**	**aOR (95% CI)**	**p**
No. of subjects	16,489	7			25			16			4			11			16			23		
**During pregnancy**																						
Any antibiotics (Yes or No), n (%)	2997 (20%)	4 (57%)	**5.3 (1.2–23.5)**	**.030**	7 (30%)	1.7 (0.7–4.2)	.229	4 (27%)	1.4 (0.5–4.5)	.537	0 (0%)	NA^#^	.986	1 (13%)	0.6 (0.1–4.6)	.592	4 (25%)	1.3 (0.4–4.1)	.636	1 (5%)	0.2 (0.0–1.6)	.126
**From birth to 1 month**																						
Any antibiotics (Yes or No), n (%)	644 (6%)	1 (20%)	3.8 (0.4–34.0)	.233	2 (13%)	2.3 (0.5–10.4)	.265	2 (14%)	2.5 (0.6–11.3)	.225	0 (0%)	NA^#^	.993	0 (0%)	NA^#^	.993	1 (9%)	1.5 (0.2–11.9)	.691	1 (7%)	1.1 (0.1–8.3)	.937
**From 1 to 12 month**																						
Infection with antibiotics (Yes or No), n (%)	3600 (38%)	3 (50%)	1.1 (0.6–2.3)	.699	3 (23%)	0.8 (0.4–1.3)	.345	8 (62%)	1.5 (1.0–2.4)	.073	1 (50%)	1.1 (0.3–3.9)	.823	2 (25%)	1.0 (0.6–1.9)	.902	6 (67%)	**1.9 (1.1–3.3)**	**.029**	6 (50%)	1.4 (0.9–2.2)	.187
No. of courses (any antibiotic), mean (SD)	0.7 (1.2)	0.8 (0.8)	1.0 (0.5–1.9)	.996	0.5 (1.2)	0.8 (0.5–1.5)	.533	1.3 (1.4)	1.3 (1.0–1.7)	.101	0.8 (1.1)	1.0 (0.3–3.1)	.998	1.5 (3.0)	1.3 (1.0–1.9)	.088	1.8 (1.8)	**1.4 (1.1–1.9)**	**.011**	1.5 (2.4)	**1.3 (1.0–1.8)**	**.037**
**From 1 to 3 years**																						
Infection with antibiotics (Yes or No), n (%)	4826 (60%)	4 (67%)	1.3 (0.2–7.3)	.737	6 (75%)	2.0 (0.4–10.0)	.394	8 (89%)	5.4 (0.7–42.8)	.076	2 (67%)	1.5 (0.6–3.8)	.426	3 (75%)	2.0 (0.2–19.3)	.546	5 (83%)	3.3 (0.4–28.6)	.270	8 (89%)	1.7 (1.0–3.0)	.061
Penicillin (Yes or No), n (%)	5352 (63%)	4 (67%)	1.2 (0.2–6.5)	.840	7 (78%)	2.1 (0.4–10.0)	.360	7 (70%)	1.4 (0.4–5.4)	.634	2 (67%)	1.2 (0.1–13.1)	.887	3 (75%)	1.8 (0.2–17.2)	.615	6 (86%)	3.6 (0.4–29.7)	.239	8 (89%)	4.8 (0.6–38.1)	.141
Non-penicillin (Yes or No), n (%)	1029 (13%)	1 (17%)	1.4 (0.2–11.9)	.765	2 (25%)	2.3 (0.5–11.5)	.305	3 (38%)	4.2 (1.0–17.4)	**.034**	0 (0%)	NA^#^	.991	1 (33%)	3.5 (0.3–38.3)	.310	2 (29%)	2.8 (0.5–14.3)	.223	1 (14%)	1.2 (0.1–9.6)	.894
No. of courses (any antibiotic), mean (SD)	1.5 (1.9)	1.0 (0.8)	0.8 (0.5–1.5)	.512	1.8 (1.5)	1.1 (0.8–1.5)	.703	2.2 (1.5)	1.2 (0.9–1.5)	.287	3.2 (4.3)	1.3 (0.9–2.0)	.145	1.8 (1.7)	1.1 (0.7–1.7)	.788	1.3 (0.6)	0.9 (0.6–1.5)	.742	2.6 (2.4)	1.2 (1.0–1.6)	.082
No. of courses (penicillin), mean (SD)	1.5 (1.8)	1.8 (0.8)	1.0 (0.4–2.8)	.987	2.0 (1.6)	1.5 (0.7–3.2)	.281	1.9 (0.7)	1.1 (0.5–2.4)	.771	1.8 (2.0)	1.3 (0.3–5.0)	.704	2.0 (0.8)	1.3 (0.4–4.2)	.661	2.6 (2.7)	1.3 (0.9–1.7)	.115	2.3 (2.4)	1.7 (0.8–3.6)	.135
No. of courses (non-penicillin), mean (SD)	0.3 (0.9)	0.5 (1.2)	1.3 (0.8–2.1)	.271	0.4 (0.7)	1.3 (0.7–2.7)	.407	0.9 (1.2)	**1.8 (1.0–3.2)**	**.036**	0.0 (0.0)	NA^#^	.991	0.7 (1.2)	1.6 (0.6–4.4)	.387	0.8 (1.5)	1.6 (0.9–3.1)	.141	0.2 (0.6)	1.0 (0.4–2.7)	.974
**From 3 to 5 years**																						
Infection with antibiotics (Yes or No), n (%)	4223 (61%)	2 (40%)	0.7 (0.4–1.6)	.436	5 (45%)	0.8 (0.5–1.4)	.495	6 (75%)	1.1 (0.6–2.0)	.670	2 (100%)	NA^#^	.320	3 (60%)	1.1 (0.5–2.3)	.770	2 (33%)	0.6 (0.3–1.2)	.149	8 (67%)	1.1 (0.7–1.7)	.684
Penicillin (Yes or No), n (%)	4813 (67%)	5 (83%)	2.4 (0.3–20.9)	.416	8 (67%)	1.0 (0.3–3.2)	.967	5 (63%)	0.8 (0.2–3.4)	.776	2 (100%)	NA^#^	.320	3 (60%)	0.7 (0.1–4.4)	.732	2 (33%)	0.2 (0.0–1.3)	.103	8 (73%)	1.3 (0.3–4.9)	.699
Non-penicillin (Yes or No), n (%)	837 (12%)	1 (17%)	1.5 (0.2–12.4)	.735	2 (18%)	1.6 (0.3–7.5)	.542	1 (13%)	1.0 (0.1–8.4)	.974	0 (0%)	NA^#^	.992	1 (20%)	1.8 (0.2–16.2)	.595	0 (0%)	NA^#^	.992	1 (11%)	0.9 (0.1–7.3)	.926
No. of courses (any antibiotic), mean (SD)	1.5 (1.8)	1.1 (1.7)	0.9 (0.5–1.6)	.617	1.5 (2.5)	1.0 (0.7–1.4)	.982	1.4 (1.2)	1.0 (0.7–1.4)	.908	4.8 (4.6)	**1.6 (1.0–2.5)**	**.048**	1.9 (2.0)	1.1 (0.7–1.7)	.639	0.5 (0.8)	0.5 (0.2–1.3)	.175	1.8 (2.3)	1.1 (0.8–1.4)	.656
No. of courses (penicillin), mean (SD)	1.7 (1.8)	2.2 (1.2)	1.5 (0.7–3.2)	.243	1.8 (1.7)	1.1 (0.7–1.7)	.809	1.4 (1.2)	0.9 (0.5–1.4)	.528	4.8 (4.6)	**1.6 (1.0–2.5)**	**.048**	1.4 (1.3)	0.9 (0.5–1.6)	.750	1.6 (3.2)	0.7 (0.3–1.3)	.250	1.3 (1.1)	1.0 (0.6–1.6)	.919
No. of courses (non-penicillin), mean (SD)	0.2 (0.8)	0.3 (0.8)	1.1 (0.4–3.0)	.812	0.3 (0.6)	1.2 (0.6–2.4)	.647	0.3 (0.7)	1.0 (0.4–2.6)	.960	0.0 (0.0)	NA^#^	.992	0.4 (0.9)	1.2 (0.5–3.4)	.675	0.0 (0.0)	NA^#^	.991	0.9 (2.7)	**1.5 (1.0–2.0)**	**.025**
**From 5 to 8 years**																						
Infection with antibiotics (Yes or No), n (%)	1450 (38%)	3 (75%)	1.9 (0.8–4.4)	.120	5 (56%)	1.4 (0.8–2.5)	.187	3 (50%)	1.4 (0.7–2.8)	.281	0 (0%)	NA	NA	3 (100%)	NA^#^	**.028**	2 (40%)	1.5 (0.7–3.1)	.267	1 (17%)	0.6 (0.2–1.6)	.289
Penicillin (Yes or No), n (%)	1562 (40%)	3 (75%)	4.5 (0.5–43.5)	.192	4 (44%)	1.2 (0.3–4.5)	.781	3 (50%)	1.5 (0.3–7.5)	.616	0 (0%)	NA	NA	3 (100%)	NA^#^	**.034**	2 (40%)	1.0 (0.2–6.0)	.996	2 (33%)	0.8 (0.1–4.1)	.744
Non-penicillin (Yes or No), n (%)	237 (6%)	3 (0%)	0.0 (0.0–0.0)	.996	16 (0%)	0.0 (0.0–0.0)	.995	2 (33%)	**7.6 (1.4–41.6)**	**.006**	0 (0%)	NA	NA	0 (0%)	NA^#^	.996	4 (100%)	5.1 (0.5–48.8)	.161	0 (0%)	NA^#^	.995
No. of courses (any antibiotic), mean (SD)	0.8 (1.3)	1.8 (1.7)	1.4 (0.9–2.1)	.142	1.4 (1.6)	1.3 (0.9–1.8)	.154	1.6 (2.0)	1.3 (0.9–1.9)	.131	0 (0%)	NA	NA	1.5 (0.0)	1.3 (0.8–2.2)	.334	2.4 (3.6)	**1.5 (1.1–2.1)**	**.011**	0.3 (0.6)	0.5 (0.1–1.9)	.309
No. of courses (penicillin), mean (SD)	0.7 (1.1)	1.5 (1.0)	1.7 (0.7–4.2)	.236	1.5 (1.9)	**1.4 (1.0–2.0)**	**.034**	1.2 (1.3)	1.3 (0.8–2.1)	.313	0 (0%)	NA	NA	2.0 (0.0)	2.7 (0.9–8.6)	.091	2.4 (3.6)	**1.7 (1.2–2.4)**	**.002**	0.5 (0.8)	0.8 (0.3–2.0)	.613
No. of courses (non-penicillin), mean (SD)	0.1 (0.5)	0.0 (0.0)	NA^#^	.995	0.0 (0.0)	NA^#^	.995	0.7 (1.0)	**2.3 (1.1–4.8)**	**.027**	0 (0%)	NA	NA	0.0 (0.0)	NA^#^	.996	2.0 (4.0)	**2.9 (1.3–6.2)**	**.007**	0.0 (0.0)	NA^#^	.995

The adjusted odds ratio is calculated in the logistic regression. Bold shows results reaching statistical significance (p-value below 0.05 and 95% CI not overlapping the null value of 1.00). NA = not applicable. # = p-value from Chi-square test

JIA = juvenile idiopathic arthritis; sJIA = systemic juvenile idiopathic arthritis; aOR = adjusted odds ratio; Oligo = oligoarticular arthritis; Poly RF- = polyarticular rheumatoid-factor-negative arthritis; Poly RF+ = polyarticular rheumatoid-factor-positive arthritis; PsA = psoriatic arthritis; ERA = enthesitis-related arthritis; Undiff. = undifferentiated arthritis

Those who fell ill with rheumatoid-factor-negative polyarthritis had a tendency toward higher antibiotic consumption during the first year of life (aOR 1.52; 95% CI 0.96–2.41, *p* = 0.073), significantly higher use of non-penicillins at the age of 1–3 years (aOR 1.83; 95% CI 1.04–3.21, *p* = 0.036) and 5–8 years (aOR 7.59; 95% CI 1.38–41.63, *p* = 0.006). Only four individuals had rheumatoid-factor-positive polyarthritis; despite this, a statistically significant increase in the intake of penicillins at 3–5 years of age (aOR 1.59; 95% CI 1.00–2.53, *p* = 0.049) was noted. Psoriatic arthritis showed a tendency toward an increase in antibiotic use in the first year of life and significantly higher use of penicillins at 5–8 years of age (*p* = 0.028).

Enthesitis-related arthritis showed a clear significant increase in the use of antibiotics, in the first year of life (aOR 1.87; 95% CI 1.06–3.28, *p* = 0.029) and at 5–8 years of age (aOR 1.52; 95% CI 1.10–2.09, *p* = 0.011), for both penicillin and non-penicillins. The group with undifferentiated arthritis, which either does not meet the criteria for any category or meets the criteria for several categories, showed significantly increased antibiotic use during the first year of life (aOR 1.34; 95% CI 1.02–1.77, *p* = 0.037) as well as for non-penicillins at 3–5 years of age (aOR 1.46; 95% CI 1.05–2.04, *p* = 0.025). In addition, there was a tendency for increased use of antibiotics at the age of 1–3 years (aOR 1.72; 95% CI 0.98–3.05, *p* = 0.061).

## Discussion

The purpose of this study was to investigate the association between infections, antibiotics and JIA. By analyzing data from a prospective birth cohort, which reflects the general population, we have found clear associations between exposure to antibiotics early in life and later onset of JIA. Our findings suggest a causal link between antibiotics and the development of JIA, which supports the hypothesis that antibiotic-induced dysregulation of the microbiome may trigger or accelerate the development of the autoimmune disease JIA in genetically predisposed children. The odds of developing JIA were three times higher in those exposed to antibiotics during childhood, compared with those not exposed. Infections during childhood, when controlling for other factors, did not show any statistically significant association with the risk of JIA. With sJIA, which is the category of JIA that most often presents initially with fluctuating fever, it is conceivable that antibiotic use would be overrepresented, which might distort the results. However, no such increased use was observed from birth to the age of 8 years in this group, only increased exposure to antibiotics during pregnancy.

Otitis (during the first year of life) and tonsillitis (at 5–8 years), often regarded as bacterial and therefore treated with antibiotics, were found more frequently in the cases than the controls, but after adjustment for potential confounders, including antibiotic treatment, this connection disappeared. This further strengthens the theory that it is antibiotic treatment itself, and not the infection, that is the environmental factor that predisposes to JIA.

The results of this study, with a clear association between exposure to antibiotics and the risk of later developing JIA, harmonize with the results of Horton et al. and Arvonen et al. [23, 24]. The data support treating different categories of JIA as separate groups for study in a subgroup analysis [30, 31]. While previous studies have not had the opportunity to do this, our material (presented in Table 4) has made this possible.

Only one JIA category showed association with maternal exposure to antibiotics during pregnancy. Interestingly, it was sJIA, which is considered an autoinflammatory disease [32]. While there are very few children with sJIA in this study, the majority had mothers who were exposed to antibiotics compared with a fifth in the controls. The mechanism behind this could possibly be the gut microbiota as previous studies have shown that microbiome diversity was affected by maternal antibiotic usage [33], but also that composition of the intestinal microbiota is different in sJIA patients compared with healthy children [34].

It is possible that the frequent exposure to antibiotics may interfere with the normal intestinal microbiota and increase the permeability of the mucosa and consequently antigen leakage, ultimately contributing to the pathogenesis of arthritis [35, 36]. In addition, antibiotics appear to affect gene expression, protein activity, and overall metabolism of the microbiota, which may directly affect immune function [37], The human microbiome might play a significant role in development of autoimmunity, as the loss of immune tolerance can be caused by microbial composition changes [38, 39]. There are several hypotheses as to how microorganisms can elicit an immune response against the host if the tolerance mechanisms fail [40]. A growing number of studies revealing the incidence of intestinal dysbiosis in various autoimmune diseases, such as inflammatory bowel disease [41], type 1 diabetes mellitus [42] and systemic lupus erythematosus [43]. There is clear evidence that the intestinal mucosa has a functional abnormality in JIA. Among other things, increased leakage of the intestinal epithelial barrier has been described [35]. In addition, in JIA patients suffering from gastrointestinal symptoms, signs of altered mucosal immunity have been observed, such as inflammatory lesions in the intestine [44], ileal lymphonodular hyperplasia [45, 46], and expression of HLA-DR in abnormal sites of the intestinal mucosa [45].Enthesitis-related arthritis (ERA) has been associated with certain intestinal microbial populations in a case-control study [47]. But other categories of JIA have also shown indications of alterations in the microbiota, as described by Arvonen et al. [48]. The intestinal microbiota develops dramatically during the first years of life [49] and stabilizes at 2–3 years of age [50]. Antibiotic exposure has a strong and sustained effect on the developing and unstable intestinal system [51, 52]. In addition, antibiotic treatment for a week has been shown to interfere with the intestinal and urinary microbiota, with changes that are measurable up to one year after the end of treatment [53–55].

The relatively low number of cases is a weakness of this study. Dropouts due to loss to follow-up are significant, but these were not found to be associated with heredity or subsequent diagnosis; therefore, skewed attrition seems unlikely. Another limitation of the study is that an claims-based assessment of antibiotic prescriptions would have been preferable instead of parent registration. While data from the Swedish Prescribed Drug Register would enable a better delimitation of the type of antibiotic, the Swedish Prescribed Drug Register with personal identity numbers (that contains all prescribed drugs dispensed at pharmacies) was established first in July 2005 and was not available at the time of initial data collection.

Our study has several significant strengths. First, the prospective study design entails minimal risk of recall and selection biases, which may occur in retrospective studies. However, this is also true for the two previous studies concerning antibiotics and JIA, whether they are register studies or claim based [23, 24]. The prospective design distributes all possible measurement biases equally between JIA cases and non-cases. In addition, the ABIS cohort has proven to be representative of the Swedish population, including current level of education [56]. Second, all cases of JIA were collected via the unique ten-digit personal identity number and ICD codes from the Swedish patient register, which has more than 99% coverage of all visits to both private and public care providers. This is a significant advantage over studies that rely solely on self-reporting. Third, we have validated all diagnoses via *the Swedish Pediatric Rheumatology Registry* and via medical records held at local health clinics and hospitals. Fourth, the availability of detailed information on important factors in early life allowed us to check for several potential confounding factors that may have affected our observed associations. To clarify the causal relationship between antibiotic exposure and JIA, our future study is focused on the microbiota during childhood and prior to diagnosis.

## Conclusions

In summary, this study shows that exposure to antibiotics early in life is associated with later onset of JIA in a large birth cohort from the general population. The relationship was dose dependent. Infections per se during childhood showed no significant association with the risk of developing JIA after adjusting for confounders. The findings suggest a causal relationship between use of antibiotics specifically and the development of JIA. Irrespective of mediating mechanisms, these results suggest that restrictive antibiotic policies during the first years of life should be advisable.

## Data Availability

The datasets are available from Johnny Ludvigsson, project leader of ABIS, on reasonable request.

## Abbreviations

JIA: juvenile idiopathic arthritis
ABIS: All Babies in South-east Sweden
ICD: International Classification of Diseases
OR: odds ratio
aOR: adjusted odds ratio
CI: confidence interval
sJIA: systemic-onset juvenile idiopathic arthritis
ERA: enthesitis-related arthritis
